# Gain control mechanisms in spinal motoneurons

**DOI:** 10.3389/fncir.2014.00081

**Published:** 2014-07-29

**Authors:** Michael D. Johnson, Charles J. Heckman

**Affiliations:** ^1^Department of Physiology, Feinberg School of Medicine, Northwestern UniversityChicago, IL, USA; ^2^Department of Physical Medicine and Rehabilitation and Department of Physical Therapy and Human Movement Sciences, Feinberg School of Medicine, Northwestern UniversityChicago, IL, USA

**Keywords:** gain, serotonin, motoneuron, spinal cord, spinal cord injury

## Abstract

Motoneurons provide the only conduit for motor commands to reach muscles. For many years, motoneurons were in fact considered to be little more than passive “wires”. Systematic studies in the past 25 years however have clearly demonstrated that the intrinsic electrical properties of motoneurons are under strong neuromodulatory control via multiple sources. The discovery of potent neuromodulation from the brainstem and its ability to change the gain of motoneurons shows that the “passive” view of the motor output stage is no longer tenable. A mechanism for gain control at the motor output stage makes good functional sense considering our capability of generating an enormous range of forces, from very delicate (e.g., putting in a contact lens) to highly forceful (emergency reactions). Just as sensory systems need gain control to deal with a wide dynamic range of inputs, so to might motor output need gain control to deal with the wide dynamic range of the normal movement repertoire. Two problems emerge from the potential use of the brainstem monoaminergic projection to motoneurons for gain control. First, the projection is highly diffuse anatomically, so that independent control of the gains of different motor pools is not feasible. In fact, the system is so diffuse that gain for all the motor pools in a limb likely increases in concert. Second, if there is a system that increases gain, probably a system to reduce gain is also needed. In this review, we summarize recent studies that show local inhibitory circuits within the spinal cord, especially reciprocal and recurrent inhibition, have the potential to solve both of these problems as well as constitute another source of gain modulation.

## Neuromodulation, a rheostat for MN excitability

It is approaching 40 years since the discovery of the powerful effects of persistent inward currents (PICs) and their ability to transform spinal motoneurons from passive conduits to active processors of incoming signals. PICs are depolarizing currents, mediated by sodium and calcium channels, primarily in the dendrites (Eckert and Lux, 1976; Schwindt and Crill, 1977; Hounsgaard and Kiehn, 1993). PICs amplify (Lee and Heckman, 2000; Hultborn et al., 2003) and prolong (Heckman et al., 2008) the effects of ionotropic synaptic inputs by producing plateau potentials and self-sustained firing and regulate the overall excitability of the cell.

PICs depend on the presence of the monoamines serotonin and norepinephrine which are produced in brainstem cells of the raphe nucleus and locus coeruleus (Hultborn et al., 2004). These cell groups send axons down to diffusely innervate all laminae and segments of the spinal cord (Björklund and Skagerberg, 1982). These so called neuromodulators act intracellularly via G-protein coupled second messengers to confer persistent behavior to dendritic calcium and sodium channels (Simon et al., 2003; Ballou et al., 2006) creating an inward depolarizing current (Carlin et al., 2000). In addition to their effects on PICs, both serotonin and norepinephrine have potent effects on the threshold of the motoneuron (Power et al., 2010). There likely also exist many other neuromodulators that influence motoneurons. Local spinal circuits can reduce the motoneuron spike afterhyperpolarization (AHP; Miles et al., 2007) whereas 5HT and NE have very little AHP effect in the adult (Li et al., 2007). Much further work is needed on neuromodulation of motoneurons; for the present, this review focuses on the effects of serotonin and norepinephrine on the PIC, which has remarkably potent effects on input-output gain of motoneurons, as explained next.

Input amplification by PICs is readily seen in electrophysiological recordings and is manifest as an increase in depolarizing current or membrane potential elicited by an excitatory synaptic input that, in the absence of PICs, would be much reduced. PIC amplification can be as great as 5-fold (Lee and Heckman, 2000). This is an essential feature for motor outputs, being one of the key mechanisms that allows spinal motoneurons to achieve firing frequencies sufficient to produce maximum voluntary muscle contractions (Binder et al., 2002). But this powerful control of intrinsic excitability of motoneurons is potentially gradable. Brainstem output patterns correlate with arousal state via the noradrenergic system and with the intensity of motor output via the serotonergic system (Rasmussen et al., 1986; Rajkowski et al., 1994, 1998). Thus by varying output from these brainstem neuromodulatory centers, motor commands can very PIC amplitude and thus vary the input-output gain of motoneurons.

It should be emphasized however this gain control has not yet been clearly demonstrated in either intact animals or in human subjects and, in fact, further experiments are needed in animal preparations to understand how much a given change in PIC amplitude increases the overall gain of the motor pool as a whole system. Nonetheless controlling gain at the motor pool makes good functional sense for the motor system as a whole. Motor output has to vary over a huge range from delicate (e.g., putting in a contact lens) to high force (moving heavy weights, high speed escapes). Varying the gain at the motor output stage allows input neurons to employ their full range of rate modulation across a wide range of motor tasks. Consistent with this possibility, chronic recordings of motor cortex neurons show a range of rate modulation that is similar at high and low forces outputs (Maier et al., 1993; Andrykiewicz et al., 2007).

PIC induced input prolongation is clearly seen in electrophysiological recordings as tail currents, plateau potentials and self sustained firing (Schwindt and Crill, 1980, 1981; Hounsgaard et al., 1988; Simon et al., 2003; Moritz et al., 2007), all present immediately after the termination of an excitatory input. Input prolongation may serve useful in the maintenance of posture, allowing brief descending commands to postural muscles in the limbs and trunk to produce persistent motor outputs (“bistable” behavior). Therefore their effect is analogous to changing the behavior of the cell to a positive integrator, for the time that the PIC is active it can sum its brief inputs to create a long lasting output. Moreover, this bistable behavior is strongest in low threshold type S motoneurons, which are heavily involved in posture. It seems reasonable to suppose that bistable behavior is routinely used for postural control, but, as for the gain control discussed above, definite data on this speculation are not yet available.

## The physical plant, a neural biomechanical link

### Inhibition provides specific control of PICs

**Figure 1:**
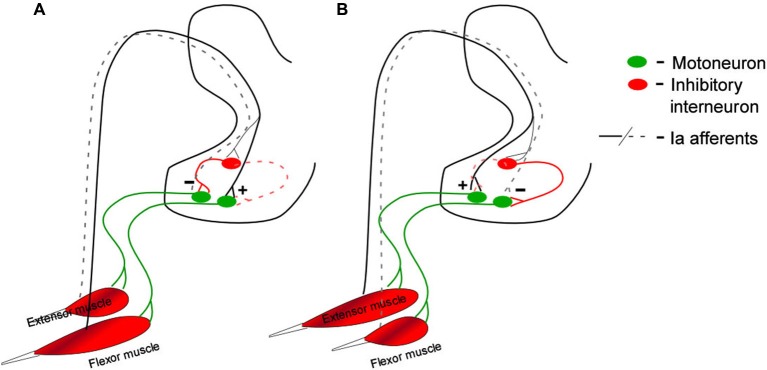
**Ia reciprocal connections to spinal motoneurons by antagonist muscle pairs**. Rotating the joint to stretch flexor muscles provides disynaptic inhibition to extensor motoneurons and direct activation of the flexor motoneuron (**A**, solid lines). The opposite rotation stretches extensor muscles providing disynaptic inhibition to flexor motoneurons and direct activation of the extensor motoneuron (**B**, solid lines). The plus and minus signs in this figure are meant to indicate net depolarization (+) and net hyperpolarization (−). During these rotations the opposite conductance change occurs at each motoneuron type: extensor muscle shortening disfacilitates extensor motoneurons (**A**, black dashed lines) contributing to their hyperpolarization, and disinhibits flexor motoneurons (**A**, red dashed lines), contributing to their depolarization. Flexor muscle shortening disfacilitates flexor motoneurons (**B**, black dashed lines, hyperplolarization) and disinhibits extensor motoneurons (**B**, red dashed lines, depolarization).

**Figure 2:**
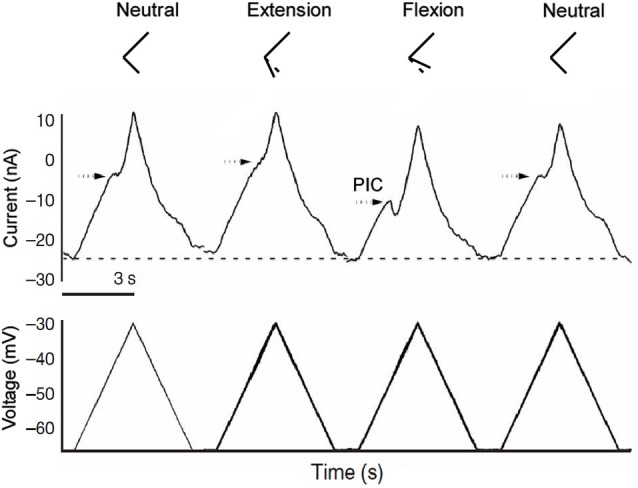
**Joint angle effects on PIC amplitude**. Joint angle positions are represented by the stick figures on top. In the midpoint position the PIC is clearly present as a downward deflection (indicating an inward depolarizing current) in the current trace. During extension, inhibition from antagonist muscle stretch greatly reduces PIC amplitude in the recorded extensor motoneuron. During flexion, antagonist muscles are shorter than in the midpoint position, providing disinhibition to extensor motoneurons revealing a large PIC.

The descending monoaminergic input to spinal MNs is diffuse and non-specific and operates through both synaptic and extra synaptic transmission (Agnati et al., 2010). Therefore judging by anatomy alone PIC effects would presumably also be broad and non-specific. Furthermore even though PIC amplitude can be globally controlled by the brainstem, the dynamics of this control are slow (Raymond et al., 2001; Hentall et al., 2006) and, since these descending tracts have no apparent somatotopy, they are not motor pool specific. Under such slow control, PIC effects such as input prolongation, which is primarily seen in low input conductance MNs and may benefit postural behaviors, could seriously interfere with the MNs ability to rapidly respond to dynamic motor commands. It has been shown that inhibition from electrical stimulation of antagonist nerves increases the threshold for plateau potentials and presumable the PICs that underlie them (Bennett et al., 1998). We have recently revealed that the Ia sensory system activated during muscle stretch associated with changes in joint angle provides a key control mechanism that confers rapid and specific modulation of PIC amplitude and effects. PICs are excellent amplifiers of excitatory synaptic inputs but can be rapidly “turned off” with synaptic inhibition (Hultborn et al., 2003; Kuo et al., 2003b; Hyngstrom et al., 2007). The Ia reciprocal inhibition system of agonist/antagonist muscle pairs is ideally constructed to provide a motor-pool-specific inhibitory control mechanism. Passively changing the joint angle in one direction (extension) stretches antagonist muscles and provides inhibition to agonist MNs (Figure 1). Changing joint angle in the opposite direction (flexion) decreases inhibition to the MN. In the case of ankle extensor MNs, antagonist muscle stretch (via ankle extension) decreases PIC amplitude by about 29% while ankle flexion, which results in a net reduction in reciprocal inhibition to extensor MNs, increases PIC amplitude the same amount (Figure 2) (more on this below). The synaptic inhibitory component of these joint rotations reduces PIC amplitude and grades their effects on MN outputs. Inhibition also modulates the electrical properties of MNs facilitating transitions between high and low excitability states. In this way the neuro-muscular physical plant provides a biomechanical control system that allows MNs to take advantage of PIC effects, which are beneficial to MN activation and output, while minimizing the potentially detrimental aspects these effects would have on motor task that involve rapidly alternating activation of muscles with opposing action.

In considering the functional effect of excitation and inhibition on the PIC, it is important to realize that the electrode is at the soma and that much of the dendritic tree is not clamped. As a result, both inhibitory and excitatory synaptic currents are more effective in changing activation of the PIC than current injected at the soma (Bennett et al., 1998; Lee et al., 2003). Previous studies (Kuo et al., 2003a; Bui et al., 2008a) and computer simulations (Bui et al., 2008b; Powers et al., 2012) suggest that the reduction in PIC amplitude by inhibition is due to both its hyperpolarizing and shunting effects. These changes likely account for the differences in PIC activation shown in Figure 2A. In other words, changes in PIC activation measured by voltage clamp at the soma are distorted by lack of space clamp of the dendritic tree. Yet, from a functional perspective, clamp at the soma is entirely appropriate and the “distortions” directly affect motor output. When a motoneuron is functioning normally, the AHP after each spike maintains the average membrane potential at a reasonably steady level—that is to say, the AHP approximately “clamps” the soma to this level (about −50 mV). Thus the effect of excitatory and inhibitory synaptic inputs on the PIC provide a reasonable estimate of how naturally evoked firing will be generated, with the important caveat that the net current to be considered should be in the voltage range for average membrane potential during repetitive firing (i.e., ~−50 mV). This functional relevance of voltage clamp current at firing level is not just an assumption. We have shown that the clamp current at firing level induced by muscle stretch (which strongly activates the PIC) provides a good prediction of the firing rate and pattern induced by an identical stretch in the unclamped state in the same cell (Lee et al., 2003). Thus it is appropriate to assess the functional effect of excitation and inhibition on PICs using voltage clamp at the soma. Nonetheless, the interaction between inputs and PICs is complicated and further work is warranted, Cutaneous, joint and muscle afferents are all activated to varying degrees during joint rotations and could potentially contribute to PIC modulation, but reciprocal inhibition by primary spindle Ia afferents dominates. This system is very sensitive to muscle length change, and the modulation of PIC amplitude by joint angle is exactly what would be predicted by Ia reciprocal inhibitory effects. We have demonstrated that PIC reduction does not occur when the tendons to antagonist muscles are cut prior to imposing joint angle changes. In fact PICs tended to be larger in the extended, flexed and midpoint joint positions in these experiments (Hyngstrom et al., 2007). The lack of PIC reduction as well as the increase in PIC amplitude in the absence of Ia reciprocal inhibition illustrates the importance of inhibitory proprioceptive inputs for modulating this intrinsic property. Finally when denervation was performed to eliminate cutaneous afferents in these experiments, results were similar to the non-deafferented condition i.e., PIC amplitudes were clearly modulated by joint angle in the absence of cutaneous inputs.

These experiments demonstrate the importance of focused reciprocal inhibition and the considerable degree of flexibility imparted by reciprocal inhibition, exerting temporally specific control over the diffuse descending neuromodulatory system. Descending brainstem inputs modulate PIC amplitude globally across all motor pools, increasing and decreasing general excitability throughout the motor system. The tightly focused inhibition from the Ia system allows specific MN behaviors to be sculpted from a slowly changing background monoaminergic state.

### Push-pull: interactions between inhibition and excitation

**Figure 3:**
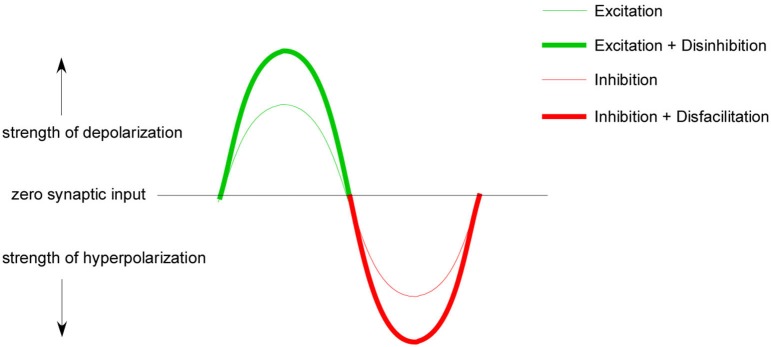
**Push-pull conceptual model**. A tonic background of excitation and inhibition is required for push-pull control. When this background is present excitation can temporally couple with disinhibition to increase excitatory gain (thick green line) and inhibition can couple with disfacillitation to increase inhibitory gain (thick red line). This conductance coupling can theoretically lead to a greater range of excitability modulation than excitation and inhibition alone (thin green/red line).

Passive joint rotation provides alternating stretch of agonist and antagonist muscles. In each rotational direction Ia afferents provide direct monosynaptic excitation to homonymous MNs and indirect inhibition to antagonist MNs (Figure 1). We have shown that reciprocal inhibition changes PIC amplitude by at least 50% (Hyngstrom et al., 2007). But there is a complementary component to both inhibition and excitation: disinhibition and disfacillitation. Synaptic inputs can interact in a number of ways. In one scheme excitation and inhibition occurs concomitantly, with one being slightly larger than the other, in what are known as “balanced networks” (Berg et al., 2007). Though metabolically expensive, balanced networks are thought to be common in the CNS. They are thought to be involved with the control of breathing (Parkis et al., 1999; de Almeida and Kirkwood, 2010) acoustic signal processing (Magnusson et al., 2008) and most notably sensory processing in the neocortex (Borg-Graham et al., 1998; Shu et al., 2003; Haider et al., 2006). Once in a balanced state, excitation and inhibition can change out of phase, creating a larger driving force for de- and hyperpolarizations, in a so called push-pull arrangement (Ferster, 1988; Grande et al., 2010; Johnson et al., 2012). Push-pull requires the presence of a tonic background of excitatory and inhibitory conductances and occurs when excitation is temporally coupled with disinhibition or when inhibition is coupled with disfacillitation (Figure 3).

**Figure 4:**
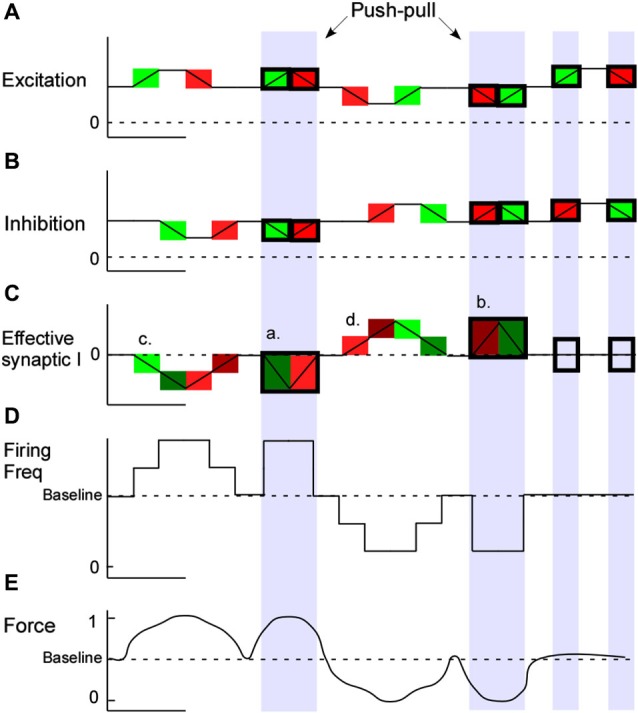
**Push-pull combinations and outcomes**. Modulating excitation and inhibition from a tonic background of each conductance should allow for greater output gain. Combinations of excitatory and inhibitory input coupling and the predicted impact on motoneuron firing and muscle force production are illustrated. In this basic model as excitatory **(A)** and inhibitory **(B)** condudtances occur in the dendrites the effective synaptic current at the motoneuron soma is illustrated in green (net depolarizing) and red (net hyperpolarizing) **(C)**. Motoneuron firing frequency changes are illustrated in **(D)** and muscle force production **(E)**. During concomitant conductance changes, push-pull effects are seen (shaded blue).

**Figure 5:**
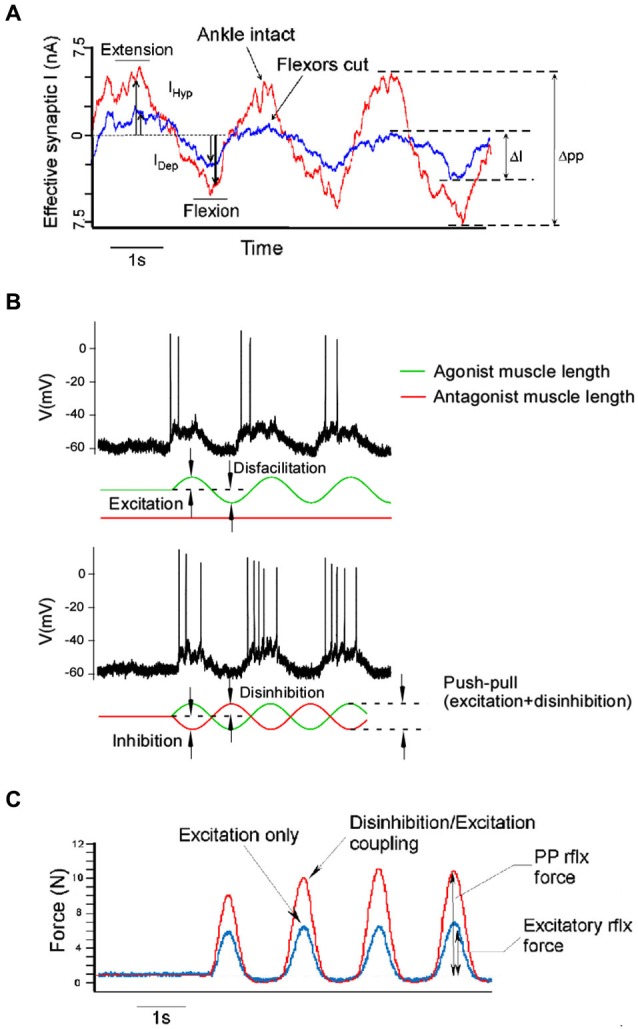
**Push-pull effects *in vivo***. Intracellular currents measured at the voltage clamped motoneuron as linear motors connected to the distal tendon of it’s agonist and antagonist muscles alternately stretch and shorten the muscles show that the push-pull configuration (tendons intact, red trace) results in greater peak to peak current amplitude than in the non-push-pull configuration (flexor tendons cut, blue trace) **(A)**. The push-pull configuration, where muscles are stretched and shortened in an alternating pattern (lower panel in **B**), also results in greater motoneuron firing rates than modulating excitation alone (upper panel in **B**). Push-pull effects manifest at the system level as well. Greater muscle force is produced when excitation is coupled with disinhibition **(C)**.

Push-pull conductance changes are reinforcing (Ferster, 1988; Conway and Livingstone, 2006) and should produce larger effective synaptic currents and larger depolarizations, greater firing frequencies and larger muscle forces than excitation alone. When opposite sign conductances modulate in phase, their effect on the neuron should cancel each other out. Combinations of excitation and inhibition and their impact on muscle force production are illustrated in Figure 4. These predictions assume purely linear interactions, which is not likely to be the case. Nonetheless our work in cat spinal MNs supports this same general pattern: coupling disinhibition with excitation produces larger excitatory currents in MNs measured in voltage clamp, larger depolarizations and higher firing frequencies in current clamp as well as greater muscle forces in unparalyzed animal experiments (Figure 5). In these same experiments we also ran trials with the inhibitory component removed by cutting the antagonist muscle tendons, effectively removing the input that provides both inhibition and disinhibition. In this altered state where excitatory inputs were modulated exclusively, MN currents, firing frequencies and muscle forces were all dramatically reduced, suggesting that Ia reciprocal inputs are superimposed on a tonic base of excitation and inhibition (Johnson et al., 2012; Figure 5).

The reciprocal organization of Ia afferents from agonist/antagonist muscle pairs is ideally suited to operate in a push-pull fashion. Push-pull is another effective strategy to increase MN input-output gain ultimately translating to increased muscle force production. Under this arrangement PIC effects are nicely regulated as well. Inhibition and excitation are smoothly modulated throughout the range of joint rotation and, from the perspective of a single MN, reverse in sign in concert with reciprocal inhibition. This allows greater depolarization in the excitatory phase, where excitation is biomechanically coupled with disinhibition, as well as strong hyperpolarizations, and therefore control of MN PICs, in the inhibitory phase where inhibition is coupled with disfacillitation. The disinhibition provided by push-pull will enhance the force output of the agonist, but if co-contraction is needed, reciprocal inhibition presumably has to be reduced and thus this mechanism will no longer be operative. Our studies involve only ankle and knee rotations, but the diverse set of descending inputs to Ia inhibitory interneurons (Baldissera et al., 1981; Jankowska, 2001) make it possible to modulate reciprocal inhibition to allow push-pull control in a wide variety of motor behaviors. Further study is required to see if push-pull control occurs at the hip or within the forelimb. The strength of push-pull effects on MN gain can be controlled by altering the background levels of each conductance.

## Spinal injury

Spinal cord injury (SCI) not only impairs motor commands but also damages descending control of spinal excitability. Paralysis, impairment and loss of function following SCI arises from loss of inputs from supraspinal structures, including those from descending neuromodulatory systems (Frigon and Rossignol, 2006). In the decerebrate cat preparation, spinal transection eliminates brainstem monoaminergic pathways as well as all other remaining descending inputs to the spinal cord caudal to the injury and thus eliminates PICs and their effects (Lee and Heckman, 2000). This leads to perhaps the most profound immediate result of spinal injury: a state of complete spinal shock where no amount of natural synaptic stimulation can bring MNs to firing threshold. In this scenario motor commands from spared pathways in incomplete spinal injury may still produce depolarizing currents in recipient MNs, but without the amplifying effect of PICs, muscle activation cannot occur. The gradual return of spinal excitability, and emergence of muscle spasms that sometimes follows, we now know, matches the time course of the re-emergence of MN PICs in animal experiments (Bennett et al., 2001a,b).

In the non-injured state spinal processing of sensory inputs produces motor outputs that are focused, reciprocal and consistent. The tightly focused Ia system that dominates MN sensory processing radically changes following SCI. Normally MNs have movement related receptive fields (MRRFs) that are joint specific. For example, in voltage clamp experiments ankle extensor MNs show strong synaptic currents during passive ankle rotation, while rotations of the hip are largely ignored. Immediately (minutes-hours) following spinal transection their MRRFs broaden and these same MNs are now strongly depolarized by rotations of the hip (Hyngstrom et al., 2008a). In this altered state the reciprocal arrangement of inputs from myotactic agonist/antagonist muscle pairs no longer dominates spinal MN behavior, in fact inputs from far away joints unrelated to the MNs muscle evoke the strongest synaptic currents. This disruption in MRRF somatotopy has, at its core, disruption of effective synaptic strengths and has the potential to interfere with reciprocal inhibitions ability to modulate MN PICs, which in the weeks following spinal injury, re-emerges.

The most likely source of this receptive field widening is acute loss of descending monoaminergic drive causing a disinhibition of polysynaptic excitatory pathways on to recipient MNs.

PICs amplify both excitatory and inhibitory inputs individually in a linear fashion. But in active networks with tonic levels of excitatory and inhibitory conductances, their combined effects display a non-linear relationship as membrane potential changes from hyper- to depolarized (Hyngstrom et al., 2008b). This non-linear relationship is actually sub linear for excitation and supra-linear for inhibition. That is, the amplifying effects of the PIC on these two separate sources of simultaneous input, which for inhibition grows stronger as the cell is more depolarized, was greater and resulted in more net inhibition in this depolarized range than what would be predicted if the inputs were applied separately and summed. The supra-linear inhibitory amplification underscores the importance inhibition plays in controlling PICs. Hence another consequence for loss of neuromodulation is disruption of the balance between the effects of excitatory and inhibitory conductances due to PIC interactions.

Though PICs and the input-output gain enhancement they impart on spinal MNs are lost in the acute stages of spinal injury, it has been shown that PICs recover within 1–5 months following complete spinal transection. This is primarily due to the emergence of constitutive activity in serotonin receptors (Murray et al., 2010). This recovery includes the plateau potentials that impart input prolongation to MNs as well as input amplification (Bennett et al., 2001a; Johnson et al., 2013). However we have shown that MNs do not recover input specificity, so they continue to have the wide MRRFs seen in acute spinal transection. As a result joint rotations not associated with their function can cause strong activations in the form of depolarizing currents (Johnson et al., 2013). These aberrant receptive fields interacting with a nearly fully recovered PIC elicit broad activation of muscles throughout the entire limb constituting, we believe, a substrate for multi-joint spasticity in the sub-acute stages of spinal injury. Our ongoing studies are focused on monitoring the changes at both the cellular, via intracellular recordings of spinal MNs, and system level, via multiple motor-unit recordings in muscles, that occur as symptoms progress through the chronic stages of spinal injury.

## Conflict of interest statement

The authors declare that the research was conducted in the absence of any commercial or financial relationships that could be construed as a potential conflict of interest.
